# P02-028 - Muckle-Wells syndrome and renal transplantation

**DOI:** 10.1186/1546-0096-11-S1-A135

**Published:** 2013-11-08

**Authors:** B Kortus-Götze, J Hoyer

**Affiliations:** 1 Nephrology, Internal Medicine, Nephrology, Marburg, Germany

## Introduction

The Muckle-Wells syndrome (MWS) is a rare inherited disease and belongs to the group of cryopyrin-associated periodic syndromes (CAPS). Recurrent fever attacks, myalgia, arthralgia, urticarial rash, headache, conjunctivitis, sensorineural deafness and a severe fatigue syndrome are the typical symptoms of MWS. Due to an unregulated production of IL1 a continuous formation of serum amyloid leads ultimately to the development of AA-amyloidosis, which is life-threatening and in some cases the fatal complication of MWS.

## Case Report

Here we report the three years follow up on a 34-year old female patient with Muckle-Wells syndrome and biopsy proven systemic AA amyloidosis and end stage renal disease. After renal transplantation therapy with canakinumab subcutaneously in a dosage of 150 mg every eight weeks was continued in combination with the immunosuppressive therapy.

## Discussion

Before and after renal transplantation the patient had a very good response to canakinumab with low activity in inflammation markers with an improved quality of life. Over the period of three years the triple immunosuppressive therapy (CSA, MMF, Prednisone) in combination with canakinumab has had no negative effect on activity of MWS and no pharmacological interactions between medication were observed. Even 3 years after renal transplantation, the patient remains an excellent kidney function without proteinuria. There are no signs of recurrence of AA-amyloidosis in the transplanted kidney.

## Disclosure of interest

None declared.
